# Predicting Functional Effects of Synonymous Variants: A Systematic Review and Perspectives

**DOI:** 10.3389/fgene.2019.00914

**Published:** 2019-10-07

**Authors:** Zishuo Zeng, Yana Bromberg

**Affiliations:** ^1^Institute for Quantitative Biomedicine, Rutgers University, Piscataway, NJ, United States; ^2^Department of Biochemistry and Microbiology, Rutgers University, New Brunswick, NJ, United States; ^3^Department of Genetics, Rutgers University, Human Genetics Institute, Piscataway, NJ, United States

**Keywords:** synonymous variants, effect predictors, variant frequency, variant functional effect, machine learning

## Abstract

Recent advances in high-throughput experimentation have put the exploration of genome sequences at the forefront of precision medicine. In an effort to interpret the sequencing data, numerous computational methods have been developed for evaluating the effects of genome variants. Interestingly, despite the fact that every person has as many synonymous (sSNV) as non-synonymous single nucleotide variants, our ability to predict their effects is limited. The paucity of experimentally tested sSNV effects appears to be the limiting factor in development of such methods. Here, we summarize the details and evaluate the performance of nine existing computational methods capable of predicting sSNV effects. We used a set of *observed* and artificially *generated* variants to approximate large scale performance expectations of these tools. We note that the distribution of these variants across amino acid and codon types suggests purifying evolutionary selection retaining *generated* variants out of the *observed* set; i.e., we expect the *generated* set to be enriched for deleterious variants. Closer inspection of the relationship between the *observed* variant frequencies and the associated prediction scores identifies predictor-specific scoring thresholds of reliable effect predictions. Notably, across all predictors, the variants scoring above these thresholds were significantly more often *generated* than *observed*. which confirms our assumption that the *generated* set is enriched for deleterious variants. Finally, we find that while the methods differ in their ability to identify severe sSNV effects, no predictor appears capable of definitively recognizing subtle effects of such variants on a large scale.

## Introduction

**Figure 1:**
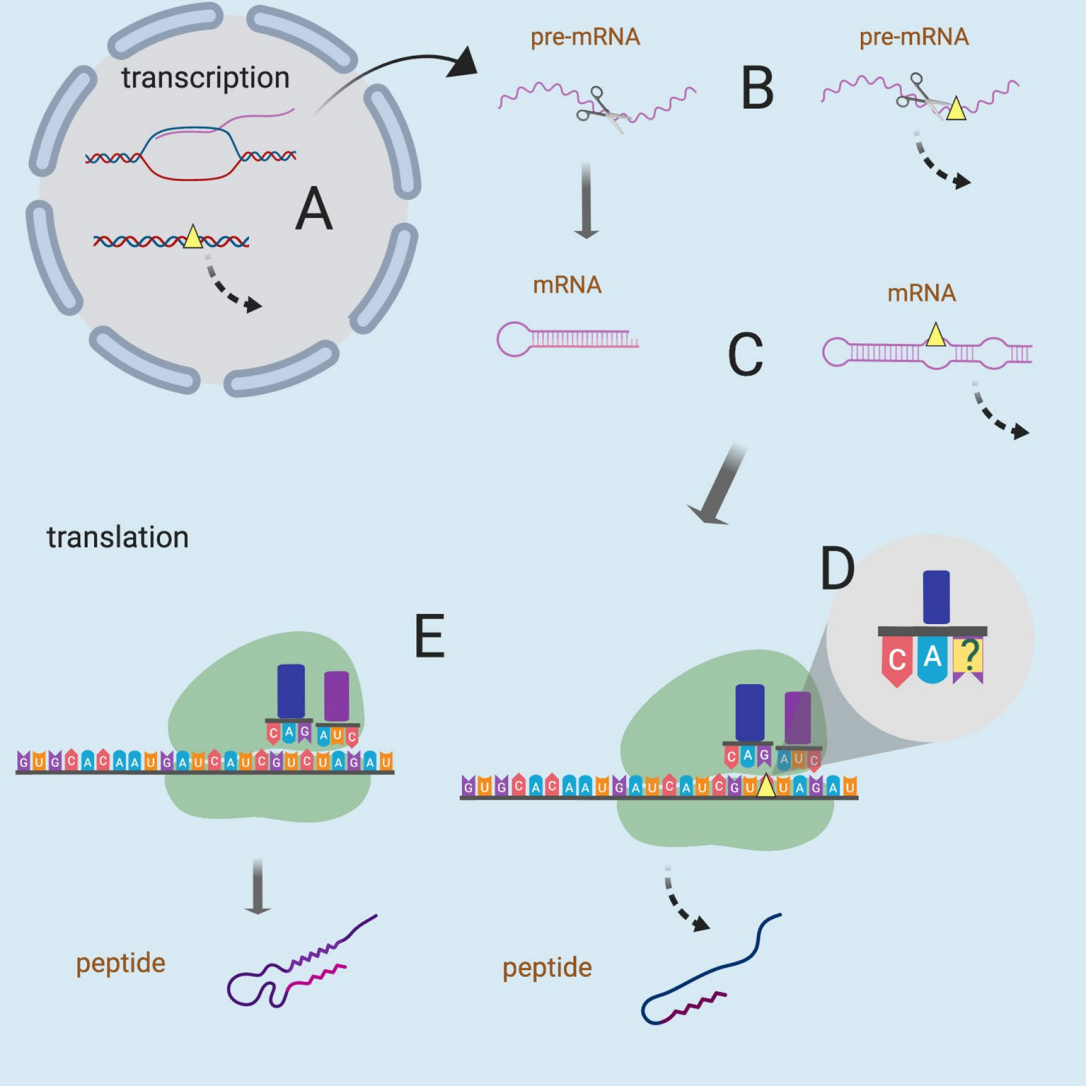
Possible mechanisms of sSNVs impact on biological function. Yellow triangles represent sSNV sites and the dashed lines indicate aberrant processes. sSNVs may affect **(A)** transcription factor binding, **(B)** splicing of pre-mRNA, **(C)** mRNA secondary structure and stability, **(D)** wobble-based tRNA binding, and **(E)** cotranslational folding (and thus the protein structure). Figure was created with BioRender.com.

The vast majority of human genomic variation is accounted for by Single Nucleotide Variants (SNVs) (Bromberg et al., 2013). The roughly 10,000 variants in the coding region of every human genome that have no effect on the resulting product protein sequence are termed synonymous SNVs (sSNVs) (Shen et al., 2013). sSNVs are a product of the degeneracy of genetic code, where amino acids may be encoded by more than one codon. The effects of sSNVs on molecular functionality of the corresponding genes/proteins are often assumed to be minimal. However, earlier studies have argued that sSNVs are as likely to be pathogenic as non-synonymous variants (Chen et al., 2010). sSNVs have been implicated in many diseases, including pulmonary sarcoidosis, attention deficit/hyperactivity disorder, and cancer (Sauna and Kimchi-Sarfaty, 2011; Supek et al., 2014). Synonymous variants can disrupt transcription (Stergachis et al., 2013), splicing (Pagani et al., 2005), co-translational folding (Pechmann and Frydman, 2013), mRNA stability (Presnyak et al., 2015) (**Figure 1**), and cause a plethora of other functionally-relevant changes. In addition, sSNVs can affect transcription and splicing regulatory factors within protein coding regions (Plotkin and Kudla, 2011), thus modulating gene expression (Shabalina et al., 2013; Boël et al., 2016). There is also evidence of evolutionary constraint on both synonymous and non-synonymous variants, which plays a role in shaping codon bias (organism or tissues-specific codon set preference) (Stergachis et al., 2013). An informative experimental approach to evaluating functional effects of sSNVs is saturation genome editing followed by protein function assays (Findlay et al., 2014; Findlay et al., 2018). Unfortunately, there are exceedingly few reports of these experiments in the literature. While there has been a concerted effort in the field to evaluate the effects of non-synonymous single nucleotide variants (nsSNVs) (Mahlich et al., 2017) for the purposes of precision medicine, as well as improving basic understanding of concepts in molecular biology, interpretation of sSNVs is severely lacking. However, considering the significant number of observed synonymous variants, their possible effects, and the dire lack of their systematic experimental interpretations, there is a compelling need for a reliable sSNV effect computational predictor.

In this paper, we review the existing sSNV-effect predictors and apply them to a dataset containing *observed* and artificially *generated* sSNVs. Since there are few experimentally-determined SNVs with deleterious effects, and those that exist have been used as training or testing sets of the predictors, the cornerstone of this study is validating our data set assumption that deleterious sSNVs are enriched in the artificially *generated* set of variants. To support this assumption, in addition to previously published work, e.g., Stergachis et al., 2013, we show that the distributions of observed sSNVs by amino acids and codons are highly non-random. We also demonstrate that existing predictor high-scoring variants are enriched among the artificially *generated* sSNVs, additionally validating of our assumption. We finally note that these predictors appear unable to definitely identify subtle effect sSNVs.

## Methodology of the Predictors

### SNV Predictors Vary by Targeted Variant Type, Training Data, and Descriptive Features

We identified from the literature four sSNV-specific effect predictors: SilVA (Silent Variant Analyzer) (Buske et al., 2013), regSNPs-splicing (Zhang et al., 2017), DDIG-SN (Detecting Disease-causing Genetic SynoNymous variants) (Livingstone et al., 2017), and IDSV (Identification of Deleterious Synonymous Variants) (Shi et al., 2019). Additionally, we considered TraP (Transcript-inferred Pathogenicity) (Gelfman et al., 2017), which addresses both synonymous and intronic variants. Specifically, 1) SilVA was trained on 33 pathogenic and 785 neutral variants from 1000 Genomes Project (1000G) (Birney and Soranzo, 2015), using conservation scores, splicing, DNA, and RNA properties, 2) DDIG-SN and IDSV used positive data from the Human Gene Mutation Database (HGMD) (Cooper et al., 1998; Stenson et al., 2003; Stenson et al., 2009; Stenson et al., 2017) and negative data from 1000G (DDIG-SN) and VariSNP (IDSV) (Schaafsma and Vihinen, 2015) as negative data for training, described using features of translational efficiency and protein properties in addition to those used by SilVA, 3) regSNPs-splicing also used HGMD and 1000G data, but it considers sSNVs only in the context of mRNA splicing and protein function, while 4) TraP was trained on positive data combining SilVA’s data with Online Mendelian Inheritance in Man (OMIM) (Hamosh, 2004) variants and negative data from control trios *de novo* variants. TraP uses transcript-affecting features, specific to intronic and synonymous variants.

As opposed to the sSNV-specific tools, more generic predictors, including CADD (Kircher et al., 2014), DANN (Quang et al., 2014), FATHMM-MKL (Shihab et al., 2015), and MutationTaster2 (Schwarz et al., 2014), evaluate synonymous, non-synonymous, regulatory and other kinds of variants. CADD was developed by training a support vector machine (SVM) to differentiate observed *vs.* simulated variants of all variant categories (Kircher et al., 2014). DANN attempts to capture nonlinear signals in (CADD-generated) variant data using a deep neural network (Quang et al., 2014). FATHMM-MKL is a Hidden Markov Model-based method integrating ENCODE (Consortium, 2012) functional annotations of SNVs to evaluate non-coding and synonymous variants (Shihab et al., 2015). MutationTaster2 (Schwarz et al., 2014) uses a naïve Bayes model trained on disease variants vs. variants from 1000G variants to evaluate all SNVs. Notably, these general-purpose predictors are heavily conservation-driven and may lack features to describe the subtle changes induced by sSNVs.

All predictors described here are machine learning-based [using random forests (RFs), SVMs, or deep neural network] and trained to predict pathogenicity, using different data and feature sets (**Table 1**). Supervised machine learning, often used for predicting variant effects, requires selecting a proper training/evaluation set, a number of relevant variant-, gene-, or disease-features, and an appropriate model for identifying feature patterns representative of variant effect/disease-association (Rost et al., 2016).

**Table 1:** Summary of sSNV-specific predictors.

Ref/Tool name	Training data	Model	Features	Performance
(Buske et al., 2013) SilVA (2013)	33 deleterious from literature, 785 neutral from one 1000 Genomes Project individual	Random forest with 1,001 trees and default number of features	26 in total • conservation • RNA properties • DNA properties • Splicing	**Dataset**: 8 DM from literature and 752 NM from literature and 1000G. **Result**: DM’s scores ranked higher than NM’s
(Gelfman et al., 2017) TraP (2017)	75 DM from literature and OMIM and 402 *de novo* NM from control trios	Random forest with 1,000 trees, each with	20 in total • Conservation • DNA properties • Splicing	**Dataset**: 66 DM and 4,418 NM from ClinVar. **Result**: AUC = 0.88
(Zhang et al., 2017) regSNPs-splicing (2017)	∼655 DM from HGMD and ∼655 NM from 1000G	Random forest with 51 trees and 35 features at each node	455 in total • Conservation • RNA properties • protein properties • splicing	**Dataset**: ∼325 DM from HGMD and 230 DM from ClinVar, ∼325 NM from 1000G and 4,535 NM from ClinVar **Result**: For HGMD vs. 1000G data, AUC = 0.91 for variants in Splice Sites and AUC = 0.82 for all others For ClinVar data, AUC = 0.85 for variants in splice sites and AUC = 0.70 for the all others
(Livingstone et al., 2017) DDIG-SN (2017)	592 DM from HGMD and 10,925 putatively benign from 1000G	Support Vector Machine with radial function kernel	54 in total (including all of the 26 features used in SilVA) • conservation • DNA properties • RNA properties • Protein properties Splicing	**Dataset**: 279 DM from HGMD and 4,945 NM from 1000G **Result**: AUC = 0.85
(Shi et al., 2019) IDSV (2019)	300 DM from dbDSM and 300 NM from VariSNP	Random forest with 500 trees and 3 features at each split	10 in total • Conservation • DNA properties • Splicing • Translational efficiency	**Dataset**: 153 DM and 5,178 NM from ClinVar **Result**: AUC = 0.87

DM, disease/deleterious mutations; NM, neutral mutations; HGMD, human gene mutation database; 1000G, 1000 genome project; OMIM, online mendelian inheritance in man; AUC, area under the ROC curve (axes in Eqn. 1).

### Available Variant Sets Are Limited in Size and Reliability

Association between genomic variants and diseases can be identified by carefully designed statistical tests, e.g., *via* Genome Wide Association Studies (GWAS) (Visscher et al., 2012). However, unequivocally identifying variants that cause disease are significantly more difficult; this is a particularly hard problem for sSNVs, which carry no corresponding protein sequence changes. Clinical or experimental validation of the causative relationships between genomic variation and disease is either infeasible altogether (as for polygenic disorders) or exceedingly difficult on a large scale due to the necessary resource and time investments. Instead, computational annotation of genomic variant pathogenicity (or simply functional effects) is a cost- and time-efficient substitute, providing a starting point for further experimental validation and discovery.

Most predictors described here (regSNPs-splicing, DDIG-SN, FATHMM-MKL, and MutationTaster2) collect variants identified as causative (positive) from HGMD. The latest version of HGMD (March 2017) comprises over 203,000 variants in over 8,000 genes, manually curated from scientific literature (Stenson et al., 2017). Despite its apparent utility, studies have questioned the reliability of HGMD data. George et al. (2007), for example, point out flaws like inconsistent mutation nomenclature and incomplete incorporation of all applicable data. Yue and Moult (2006) note that some mutations in HGMD are named causes of monogenic disease but are not fully penetrant, while Bell et al. (2011) question disease annotation of recessive variants. In a study of 1,000 exomes, Dorschner et al. (2013) note that only 16 of 585 of HGMD disease-causing variants were actually pathogenic, while in a subsequent study with 6,503 individuals, none of the identified 615 HGMD disease-causing variants were pathogenic (Amendola et al., 2015). Other studies (Xue et al., 2012; Cassa et al., 2013) have shown that many disease-causing variants in HGMD are present in the relatively healthy 1000G individuals (Birney and Soranzo, 2015).

Other sources of positive training/testing data, including OMIM (used by TraP) and ClinVar (used by TraP, regSNPs-splicing, IDSV, CADD, MutationTaster2, and FATHMM-MKL) (Landrum et al., 2014), appear no more reliable. Notably, there is considerable inconsistency between the HGMD and OMIM (George et al., 2007). ClinVar’s entries from different sources often conflict between themselves (Landrum and Kattman, 2018), as the reliability of ClinVar’s data curation and workflow of medical interpretation has not been proven (Bauer et al., 2018). Substantial discordance between ClinVar and laboratory test results has also been reported (Gradishar et al., 2017).

Mutation databases vary drastically (George et al., 2007), not in the least because of experimental interpretation differences; e.g., roughly 17% of the variant effects reported by different laboratories carry contradictory clinical significance (Rehm et al., 2015). Labels of pathogenicity are not fixed, switching from disease to benign and back as evidence accumulates (Shah et al., 2018). As these binary labels also do not provide a quantitative measure of risk (Shah et al., 2018) or penetrance, the term “disease-causing” should be used with caution. One key problem in the field, and a reason for many of the above data limitations, is the absence of a gold standard for identifying disease-causing variants (Dorschner et al., 2013). Moreover, even the “silver-standard” available annotations are far and few between. In fact, while there are many known pathogenic nsSNVs, there are currently much fewer known pathogenic sSNVs available: dbDSM (Wen et al., 2016) (including those from ClinVar, PubMed, NHGRI GWAS catalog (Welter et al., 2013), etc.) contains 1,289 pathogenic sSNVs, and HGMD contains roughly 900 pathogenic sSNVs (Livingstone et al., 2017). Arguably, this number is too small to build a generalizable model for evaluating tens of millions of the possible synonymous variants in human genome. Note that an additional problem is the absence of a true negative set of variants, i.e., those that have been verified to have no effect on protein function or no relationship to some disease (Bromberg et al., 2013).

### Use of Allele Frequency to Approximate Variant Effect

SilVA was trained on 33 experimentally defined deleterious and 785 assumed neutral (observed in 1000G) variants. While the former set was very stringently selected, this small number of samples could hardly produce a generalizable model. Other predictors use less well curated data from available databases, but as such run into a problem of reliability. To supplement the lack of experimentally annotated variation, variant population frequency had been suggested as a sign of effect/pathogenicity; i.e., it is generally assumed that disease/effect variants are of low allele frequency (Gibson, 2012), although the precise threshold for “low” is unclear. Predictors (CADD, DANN, FATHMM-MKL, SilVA, regSNP-splicing) often filter out effect variants of higher frequency and/or neutral variants of lower frequency. CADD and DANN training data, for example, contains simulated human variants, appearing after human-chimpanzee divergence, labelled as the effect group (depleted by natural selection) and observed fixed or nearly fixed derived alleles as neutral (Kircher et al., 2014; Quang et al., 2014). Note although simulated variants are likely enriched in deleterious variants, and CADD scores have been shown useful in prioritizing variants in clinical settings (Amendola et al., 2015; Nakagomi et al., 2018; Van Der Velde et al., 2015), it is difficult to directly link the CADD predictions to pathogenicity (Kircher et al., 2014).

Allele frequency, however, is not necessarily correlated with variant effect, particularly when effect being considered is “function change” not “disease.” In an earlier study, we found that common [minor allele frequency (MAF) > 5%] non-synonymous variants were more often predicted to have a functional effect than rare (MAF < 1%) ones (Mahlich et al., 2017). Here a high-frequency allele may be beneficial/advantageous and on the way to becoming common, or slightly deleterious and on its way out (Bromberg et al., 2013). Moreover, trivially, allele frequency estimated from the sequenced genomes may be subject to change as the number of samples increases. Thus, 1) low allele frequency is not equivalent to having an effect and 2) although high frequency alleles are unlikely to be disease causing, they may have some impact. Additionally, and perhaps most fundamentally, note that the currently observed SNVs are unlikely a complete set of naturally occurring variants, i.e., many SNVs may be yet unseen.

### Features Used Vary From Predictor to Predictor

A variety of features have been considered by predictors as described below. Note that the number of features used in existing predictors ranges from 26 (SilVA) to 1,281 (FATHMM-MKL).

#### Conservation

Evolutionary conservation, derived from multiple sequence alignments (MSAs) of homologous sequences (Niroula and Vihinen, 2016), is perhaps the most extensively used feature of variant-effect predictors. Commonly used DNA conservation scoring algorithms include GERP (Cooper et al., 2005), phastCons (Siepel et al., 2005), and PhyloP (Pollard et al., 2009) scores. GERP (Genomic Evolutionary Rate Profiling) is a statistical method identifying genomic constrained elements from MSAs. GERP uses a statistical model estimating species divergence times (Hasegawa et al., 1985) and a structural expectation maximization algorithm for phylogenetic inference (Friedman et al., 2002); the later GERP++ is a faster version of the original (Davydov et al., 2010). phastCons fits MSAs to phylogenetic hidden Markov models to identify conserved elements (Siepel et al., 2005). The major difference between phastCons and GERP is that the former models the size and distribution of conserved elements within an MSA, while the latter first individually assesses the conservation at a locus and then searches for clusters of highly conserved loci (Chen et al., 2010). PhyloP combines statistical tests and GERP to detect conservation and acceleration in nucleotide substitution rates (Pollard et al., 2009). All variant effect predictors use at least one of these conservation scoring techniques (**Tables 1**, **2**). DDIG-SN also additionally uses protein conservation as conserved protein positions are often structurally important (Ng, 2003), suggesting possible misfolding due to decreased rate of translation at the relevant codon. Similarly, sSNVs may lead to mistranslation (Kramer and Farabaugh, 2006; Kramer et al., 2010; Komar, 2016) resulting in amino acid substitutions—a particularly problematic occurrence at conserved protein positions.

**Table 2:** Summary of generalized SNV predictors.

Ref/Tool name	Training data	Model	Features	Performance
(Kircher et al., 2014) CADD (2014)	13,141,299 SNVs, 627,071 insertions, and 926,968 deletions from simulated and observed variant sets	SVM with linear kernel	63 in total • Conservation vVariant consequence • DNA features • Other	**No testing of synonymous variants**
(Quang et al., 2014) DANN (2014)	13,302,220 observed variants; 13,302,220 simulated variants selected from CADD data	Neural network with 3 1,000-node hidden layers	63 features from CADD	**All types of variants, amount of sSNVs not stated** **Dataset**: 162,777 observed and 162,777 simulated variants (including synonymous variants). **Result**: Overall accuracy = 0.66
(Shihab et al., 2015) FATHMM-MKL (2015)	1,073 coding DM from HGMD and 1,073 coding NM from 1000G for 10-feature-group model; 3,000 coding DM from HGMD and 3,000 coding NM from 1000G for 4-feature-group model	Multiple kernel learning	1,281 in total • Conservation • DNA properties • Other	**Coding variants, amount of sSNVs not stated** **Dataset**: 5-fold cross-validation from training data **Result**: AUC = 0.93 and 0.91for 10-feature-group model and 4-feature-group model, respectively
(Schwarz et al., 2014) MutationTaster2 (2014)	122,238 DM from ClinVar and HGMD; 6,807,269 NM from 1000G	Bayesian classifier	∼ 7 (not explicitly stated) in total • Conservation • DNA properties • Splicing	**No testing of synonymous variants**

DM, disease/deleterious mutations; NM, neutral mutations; HGMD, human gene mutation database; 1000G, 1000 genome project; AUC, area under the receiver operating characteristic curve.

Conservation is a very important signature of variant effect. For example, for ClinVar’s missense dataset the solely-conservation-based component of CADD, GerpS (a derivative of GERP++), as well as PhastCons and PhyloP, attained ROC AUCs (area under the receiver operating characteristic curve) of over 0.82, while CADD’s ROC AUC was only slightly higher (0.93) (Kircher et al., 2014). In FATHMM-MKL’s cross-validation on coding variants, its ROC AUCs was = 0.93 while the ROC AUCs for conservation scores alone was = 0.91 (Shihab et al., 2015). Similar results are observed for DDIG-SN (DDIG-SN’s ROC AUCs = 0.85, PhyloP’s ROC AUCs = 0.76) (Livingstone et al., 2017) and TraP (TraP’s ROC AUCs = 0.88, GERP++’s ROC AUCs = 0.87) (Gelfman et al., 2017) datasets. These results suggest that over billions of years of evolution, nature’s laboratory has tested and discarded most of the detrimental variants. However, it is important to note that functional tuneability, i.e., development of new or environment-specific versions of functions is an ongoing process, which requires the presence of variants in positions of all levels of conservation, in any given snapshot of a population (Miller et al., 2017; Miller et al., 2019).

#### DNA Properties

The DNA properties describing the biological effects of sSNVs include but are not limited to localization to transcription factor (TF) binding sites, overall GC content of genes and genomes, and CpG island locations (cytosine followed by guanine in 5’ to 3’ direction). In more detail: many studies have shown that coding exons can serve as regulatory elements for transcription (Lang et al., 2005; Khan et al., 2012); i.e., roughly 15% of the human genome codons both code for amino acids and specify TF recognition (Stergachis et al., 2013). Thus, synonymous variants in TF-relevant codons can affect TF binding and alter gene transcription rates. Exonic and the flanking intronic region GC architectures can affect DNA methylation and exon recognition (Gelfman et al., 2013). Additionally, CpG sites often host DNA methylation (Bernstein et al., 2007), playing an important role in gene transcription (Gelfman et al., 2013). As mutation rates at CpG dinucleotides are an order higher than at other sites (Nachman and Crowell, 2000), sSNVs can thus alter methylation patterns by disrupting site-specific GC architectures.

All predictors covered in this manuscript, except regSNPs-splicing, incorporate one or more of these DNA properties (**Tables 1**, **2**).

#### RNA Properties

*Codon bias*. The preference (frequency of use) of particular codons by specific organisms or tissues is termed codon bias. Codon bias correlates with and informs gene expression levels (Coghlan and Wolfe, 2000; Carbone et al., 2003; Dos Reis et al., 2003; Boël et al., 2016; Komar, 2016), translation rate (Sørensen et al., 1989), as well as protein structure (Zhou et al., 2009) and cotranslational folding (Pechmann and Frydman, 2013; Buhr et al., 2016). There are many different metrics describing codon bias including codon adaptation index (Sharp and Li, 1987), synonymous codon usage order (Angellotti et al., 2007), relative synonymous codon usage (Sharp and Li, 1987), etc. Surprisingly, only SilVA and DDIG-SN have considered codon bias as a factor in their models (**Table 1**).

A related factor governing translation rate is the supply of tRNA during translation. Note that tRNA concentrations are different across organisms and that some organisms lack certain tRNA altogether, supplementing the necessary functionality *via* third position wobble (Novoa et al., 2012). It is hypothesized that codon composition in coding regions coevolved with tRNA abundances to reach the desired translation rates (Plotkin and Kudla, 2011). tRNA adaptation index (tAI) (Reis et al., 2004), used only by IDSV (**Table 1**), is a measure aimed to describe codon bias from the perspective of tRNA supply and demand.

A potentially important feature also missing from all predictors is codon autocorrelation. In codon autocorrelated sequences, same codons follow each other in sequence, i.e., sequence AAABB is more autocorrelated (less anticorrelated) than sequence ABABA, where A and B are two codons of the same amino acid (Cannarozzi et al., 2010). Autocorrelated yeast transcripts are translated faster than anticorrelated ones (Cannarozzi et al., 2010) and many prokaryotes modulate translation through codon correlation (Guo et al., 2012). Thus, using codon correlation may help characterizing sSNV effect.

*mRNA structure, stability, and abundance*. sSNVs can alter mRNA secondary structure, thus impacting translational efficiency and mRNA decay rate (Hunt et al., 2014), which, in turn, impacts protein production (Komar, 2016) and abundance (Maier et al., 2009). mRNA sequences are more stable than random collections of nucleotides (Seffens, 1999), suggesting that mRNA stability is evolutionarily selected to accommodate sufficient levels of translation before decay. The secondary structure of mRNAs harbors conserved elements (Meyer, 2005) and is tightly interwoven with GC content and codon usage. In fact, an earlier study found that 26% of the expressed genes display differential mRNA stability across individuals (Duan et al., 2013). In these genes, higher GC3 (G or C at the third position of the codon) percentage correlated with higher mRNA stability. This finding is in line with the fact that among the different SNVs, G and C alleles generally result in higher mRNA stability than A and T alleles (Duan et al., 2013). Furthermore, stability is enhanced in mRNA sequences enriched in optimal codons corresponding to tRNAs of higher concentrations (Presnyak et al., 2015).

A number of *in silico* tools have been developed to predict the mRNA structure and stability, including mFold (UNAFold) (Zuker, 2003; Markham and Zuker, 2008), remuRNA (Salari et al., 2012), KineFold (Xayaphoummine et al., 2005), and RNAfold (ViennaRNA package) (Hofacker, 2003). Note, however, that RNA molecules are very thermodynamically flexible and can take on many possible structures. Thus, the predicted RNA structure and its stability depend on the pre-set prediction strategy, which can be aimed to find the minimum free energy structure, the structure closest to other possible structures, or to maximize expected prediction accuracy, which is difficult for RNAs longer than 500 nucleotides (Lorenz et al., 2016). Consequentially, the prediction of RNA structure and stability is inherently uncertain. Among all the sSNV predictors, only SilVA and DDIG-SN use predictive tools to compute the variant-induced changes of energy and structures in pre-mRNA and mature mRNA sequences (**Table 1**).

Note that sSNVs, as well as other variant types (Shah and Gilchrist, 2010), are particularly relevant to functionality of highly expressed genes. Thus, the Genotype-Tissue Expression (GTEx) project’s database containing large-scale human tissue-specific gene expression data (Lonsdale et al., 2013) can be used to establish genes that are likely to manifest sSNV effect. However, none of the predictors described here use expression information to inform their effect predictions.

#### Splicing Properties

mRNA splicing is a major predictive feature in some predictors, especially regSNPs-splicing and IDSV. It is estimated that up to 15% of disease SNVs cause aberrant splicing (Krawczak et al., 1992). sSNVs can impact exonic splicing enhancers (ESEs) and silencers (ESSs), i.e., short DNA sequence motifs that promote or suppress splicing of pre-mRNA by binding to SR proteins (proteins with long repeats of serine and arginine) (Wang and Burge, 2008). Moreover, sSNVs can change the affinity of pre-mRNA to spliceosomes, leading to false recognition of exon-intron boundaries and producing abnormal mRNAs and dysfunctional proteins (Bali and Bebok, 2015). Taken together, the sSNVs’ potential of disrupting splicing is the likely reason for slower evolution at within-ESE sites (Parmley, 2005).

Predictors describe the potential impact of sSNVs on splicing by relying on the identified putative ESE and ESS motifs. Identification of these motifs and the corresponding splicing regulatory proteins has been an ongoing experimental and computational effort (Wang and Burge, 2008; Shepard and Hertel, 2009); identified motifs and regulatory proteins are available *via* public repositories (Desmet et al., 2009; Giulietti et al., 2013; Xing et al., 2016). Tools such as SPANR (Splicing-based Analysis of Variants) (Xiong et al., 2015), have also been developed to predict the splicing effects of SNVs. Splicing is considered by all sSNV-specific predictors, although represented *via* different values.

#### Protein Properties

One often overlooked aspect in evaluating sSNV effect is the protein structure. Rare codon variants of frequent synonymous codons may slow down the translation rate due to low concentration of tRNAs, slow or stop the elongation of the peptide chain (Zhang et al., 2009), and influence co-translational folding (Kimchi-Sarfaty et al., 2007; Pechmann and Frydman, 2013). Cotranslational folding is closely related to the formation of protein secondary and tertiary structures (Holtkamp et al., 2015); alpha-helix formation can occur in the ribosomal tunnel (Komar, 2009), while tertiary structure formation may take place before the protein completely exits the ribosome (Zhang and Ignatova, 2011). Translationally fast codons are enriched for alpha helices, while beta strands and coil regions prefer translationally slow codons (Thanaraj and Argos, 1996). Optimal codons are enriched in buried and structurally important sites but are negatively correlated with solvent accessible sites (Zhou et al., 2009). Pathogenic sSNVs are generally enriched within the buried sites, intrinsic disorder regions, and alpha-helices, as well as in exons overlapping with known or predicted protein family domains (Zhang et al., 2017). These findings suggest that protein structure should be considered when modelling the effects of sSNVs. However, only regSNPs-splicing and DDIG-SN predictors incorporate protein structural information (**Table 1**).

## Evaluation of the Predictors

### Collecting the Evaluation Data Set

sSNV effect predictor evaluation is hampered by three major problems: 1) there is no clear definition of neutral and effect variants and 2) available neutral/effect experimental evaluations are limited, and 3) most have been used in predictor development. Here, we created our own data set of variants for evaluation purposes as follows: we collected the *observed* sSNVs [all non-singleton sSNVs that have been observed in either 1000G, ExAC (Lek et al., 2016), or gnomAD (Karczewski et al., 2019)] and the *generated* sSNVs (all possible sSNVs in human genes, excluding *observed* and singleton sSNVs); we thus extracted 1,362,607 *observed* and 24,008,961 *generated* sSNVs. For evaluation purposes, we randomly selected 1,362,607 *generated* variants from our set to create a balanced *observed/generated* variant *Test set* (details in **Supplementary Material**).

There are multiple equally valid reasons for why nearly 95% of all possible sSNVs are not *observed*; the most obvious ones are technical, i.e., insufficient data or sequencing technology bias, and evolutionary, i.e., purifying selection, genetic drift, and genetic hitch-hiking (Smith and Haigh, 1974). As per the latter, we assume that drastically deleterious variants, which would be eliminated on a population scale due to purifying selection, are significantly more frequent in the set of *generated* sSNVs than in *observed* ones. However, the former suggests that we may have simply not (yet) sequenced many of the un-observed *(generated)* variants, which are actually equivalent in potential effect to *observed* ones. Notably, since a large proportion of discovered sSNVs are singletons (Lek et al., 2016), an equivalent proportion of similarly neutral or mild-effect variants can likely be found on the other side of the “sequencing barrier,” i.e., they have yet to be sequenced. Moreover, different categories of variants vary in the likelihood of being observed. For example, according to the ExAC project, the discovery of CpG transitions (C- > T, where C is followed by G) is likely close to saturation, while additional transversion and non-CpG transitions are yet to be identified (Lek et al., 2016).

We observe that 1) most of the large effect variants are likely in the *generated* set and either 2a) they make up much of that set, i.e., the *generated* set contains mostly effect variants, or 2b) there are relatively few of them, i.e., the distribution of effect and neutral variants is roughly equivalent across the *generated* and *observed* variants. Note that if (2a) is true, we expect that a precise and sensitive sSNV effect predictor should be able to differentiate the *observed* sSNVs from the *generated* ones, while (2b) would mean that the same predictor would produce similar effect distributions.

Note that our *Test set* data are collected in a somewhat similar, but ultimately very different, way as CADD’s (and DANN’s) training data. CADD’s observed variants are the fixed or nearly fixed alleles at sites where human genes are different from the inferred human-chimpanzee ancestor and thus may encompass our excluded *observed* singletons. CADD’s simulated variants follow an estimated *de novo* mutation rate since human-chimpanzee divergence, and thus are a subset of all our variants, including *generated, observed*, and singletons. Importantly, even with down-sampling of *generated* variants to create a balanced set, our *Test set* is much larger (∼2.8 million) and more broadly defined than CADD’s strictly curated training set (∼100,000).

**Figure 2:**
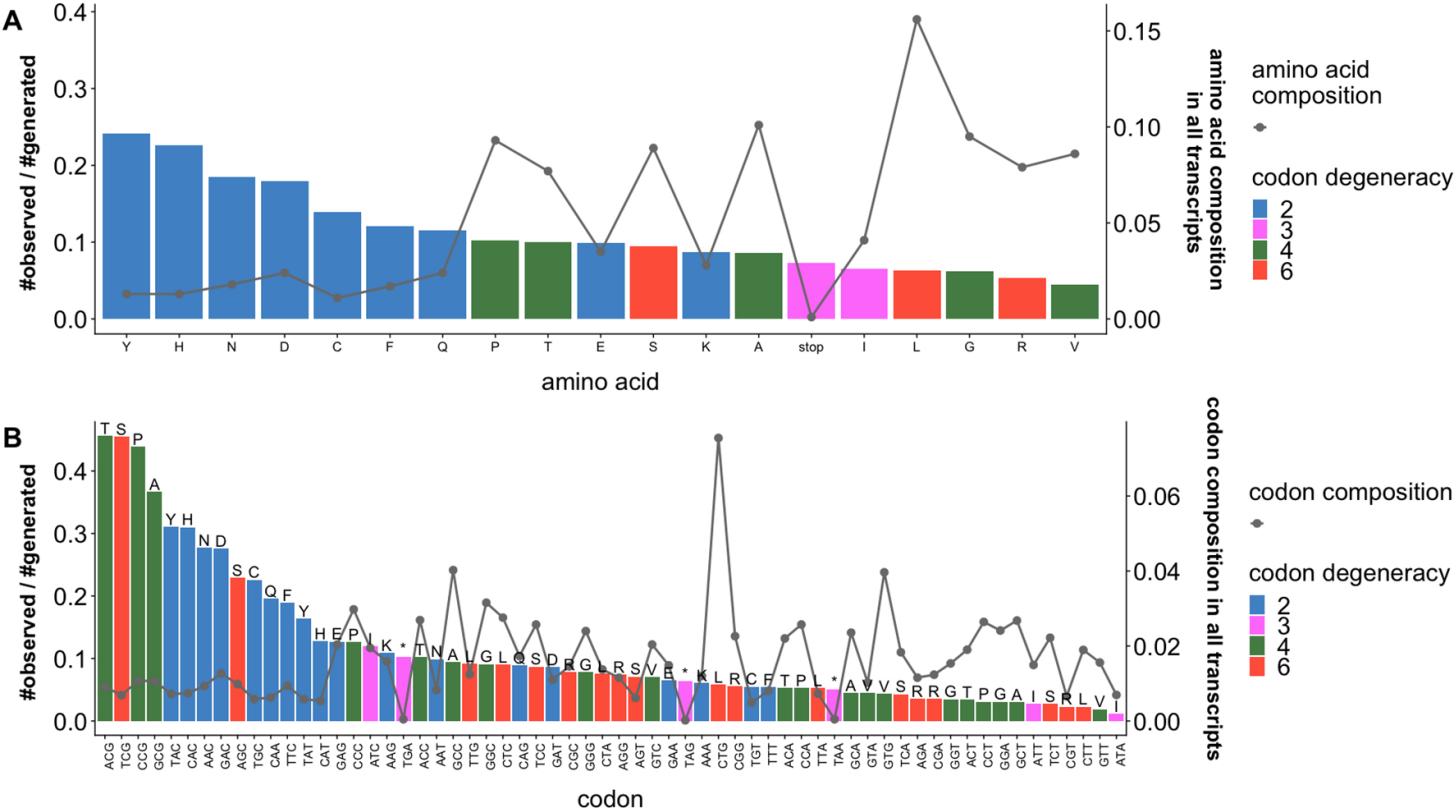
Ratios of *observed* and *generated* sSNVs vary across codons and amino acids. Ratios of *observed* to *generated* sSNVs (barplot, left axis) affecting specific **(A)** amino acids and **(B)** codons in the human transcriptome differ. Lines (right axis) in plots indicate the fractions of **(A)** amino acids and **(B)** codons (“*” is a stop codons). Trivially, 2-codon amino acids are generally enriched for *observed* sSNVs, while higher degeneracy codons are depleted. However, there is a significant difference between the most and least frequent 2-codon amino acid sSNVs. Codons with an NCG pattern (N = any nucleotide) are most often affected by sSNVs. On the other hand, codons with a CGN pattern (also CpG) are relatively rarely affected. Note that amino acid degeneracy is correlated with % composition, although a single codon is often responsible for coding most of each of these amino acids (e.g. Leucine CTG and Valine CTG).

We calculated the enrichment of *observed* sSNVs relative to *generated* sSNVs separately by amino acid (**Figure 2A**) and codon (**Figure 2B**) type. We observe that the distribution of naturally occurring sSNVs is non-random across amino acids and codons. Thus, over a fifth of all tyrosine (Y) and histidine (H) codons in our genome is affected by sSNVs, as compared to roughly 8% of lysine (K) codons. Curiously, the most mutated codons are threonine ACG, serine TCG, and proline CCG (> 43% of each is affected by an sSNV) and alanine GCG (37%). Thus, the CG end-of-codon nucleotide pair seems to indicate least stable codons. On the other hand, the isoleucine ATA codon is almost never mutated (∼1%), suggesting that it is preferentially maintained as error free. Moreover, the enrichments of observed sSNVs by amino acids (or codon) are not proportional to the abundance amino acids (or codon) in human transcriptome. The amino acids (e.g., Y, H, N, D) and codons (e.g., ACG, TCG, CCG, GCG, TAC, CAC) with high enrichment of *observed* sSNVs are those of low abundances. This decidedly non-random distribution of variants across codons and amino acids strongly suggests that our *generated* and *observed* variants are likely indeed different from the evolutionary, and thus likely effect, perspective.

### Predictors Do Not Distinguish *Observed* and *Generated* sSNVs

To the best of our knowledge, our collection of tools (CADD, DANN, MutationTaster2, FATHMM-MKL, SilVA, TraP, DDIG-SN, regSNP-splicing, and IDSV) make up a complete set of publicly available methods for sSNV analysis. We first evaluated (**Figure S2**) the ability of all predictors (except regSNP-splicing, which was not functional at the time of writing) to differentiate 50,000 *observed* and 50,000 *generated* sSNVs (**Supplementary Materials**). We did not include IDSV for more further analysis because its performance was similar to that of other predictors and it was not available for running it locally or online for the entire set of our variants. Unfortunately, we also had to exclude MutationTaster2, which experienced server problems when running large batches of data.

**Figure 3:**
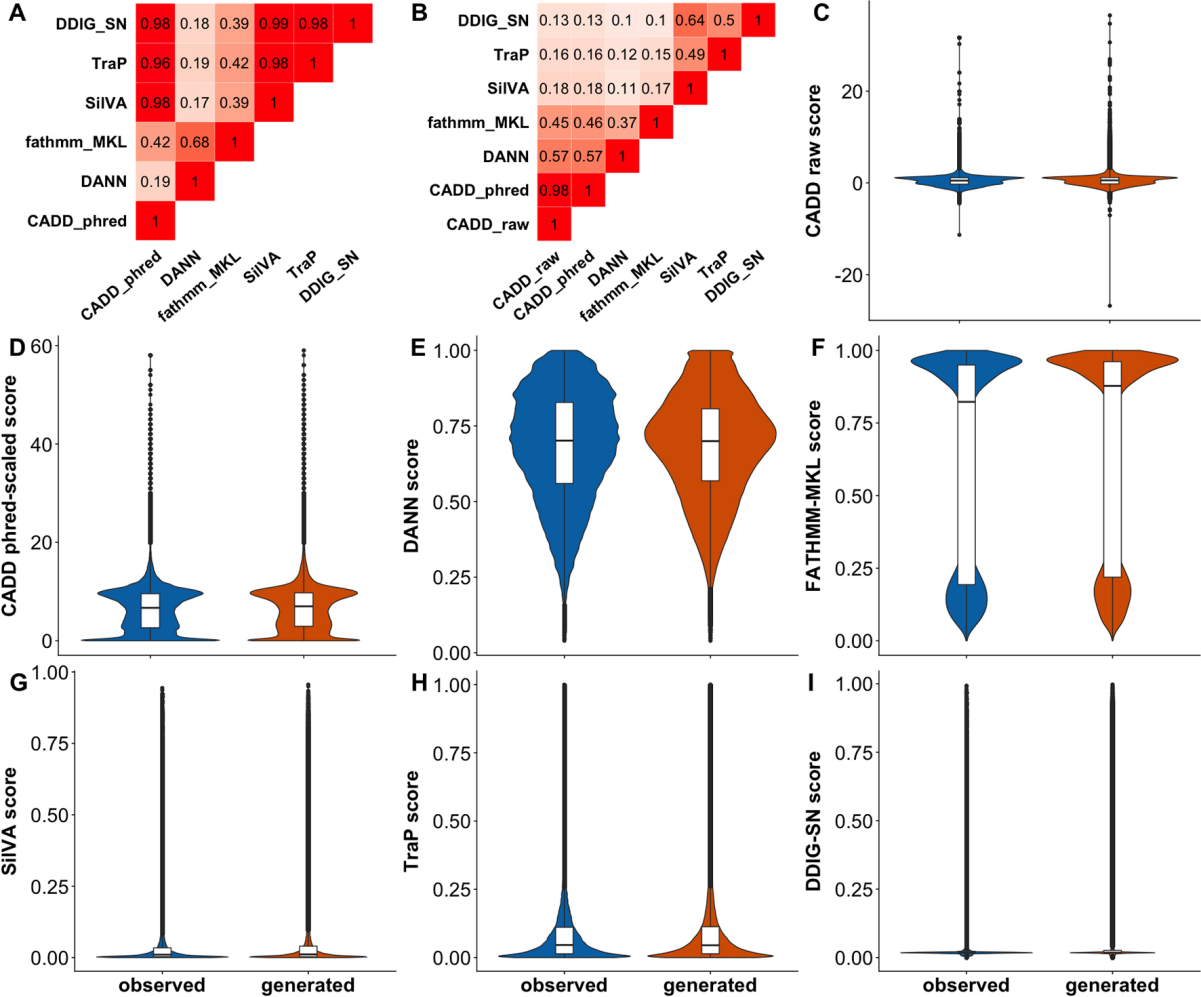
Predictor scores correlate somewhat, but do not differentiate *observed* vs. *generated* sSNVs. Panel **(A)** shows the amount of agreement (i.e., FCBP) for any pair of predictors. High FCBP values indicate that two predictors agree in assigning binary (neutral/deleterious) predictions to variants. Panel **(B)** shows the Pearson correlations among the prediction scores. **(C**–**I)** Violin/box plots of prediction score distributions across predictors: CADD raw, CADD phred-scaled, DANN, FATHMM-MKL, SilVA, TraP, and DDIG-SN, respectively.

We used CADD, DANN, FATHMM-MKL, SilVA, TraP, and DDIG-SN to make predictions for our complete variant *Test set*. We calculated the fraction of consensus binary predictions (**Figure 3A**) (FCBP; i.e., the number of predictions agreed upon) for all pairs of predictors and the correlation between scores (**Figure 3B**). As per CADD creators (https://cadd.gs.washington.edu/info), it is hard to threshold its raw scores, while the recommended neutral/deleterious cutoff for phred-scaled scores is 15. For the rest of the predictors, we used 0.5 as the binary threshold (> 0.5 is deleterious). We observed (**Figure 3A**) that the CADD and other sSNV-specific predictors agree with each other because their scores are mostly low (**Figures 3F–H**). Scores from general-purpose predictors do not have high correlation with sSNV-specific predictors. Meanwhile, DANN and FATHMM-MKL did not agree with others or between themselves. This lack of agreement across the *Test set* indicates that, in the best case, predictors are orthogonal, correctly identifying a different subset of variants each or, in the worst case, they are mostly unable to recognize effect. Curiously, for each predictor, the distributions of sSNV scores of *observed* and *generated* variants were very similar (**Figure 3**), i.e., predictors disagreed between themselves and with our dataset labels. Note that since the data is large, statistical tests to establish their difference could easily achieve significance and may not be meaningful (Kim and Bang, 2016). Instead, we directly evaluated predictor ability (**Table 3**) to differentiate the two types of variants using ROC AUCs. ROC curve is a plot of true positive rate (TPR) against false positive rate (FPR), which are computed with true positive (TP), false negative (FN), and false positive (FP) (Eqn. 1). No predictor was able to accurately differentiate *generated* and *observed* variants well. To evaluate the variation of different predictors introduced by the sampling of the *generated* set, we also subsampled the *observed* and *generated* sets for 20 times (each with 100,000 samples) and calculated the resulting standard errors of ROC AUCs (**Table 3**).

(1) $$TPR=\frac{TP}{TP+FN},\;FPR=\frac{FP}{FP+TN}$$

**Table 3:** AUCs of the predictors on sSNVs and nsSNVs.

	*Observed* vs. *generated* sSNVs		*Observed* vs. *generated* nsSNVs
	AUC on *Test set*	Average of AUCs ±SD *	
CADD raw score	0.518	0.517±0.0012	0.564
CADD phred-scaled score	0.518	0.518±0.0013	0.564
DANN	0.506	0.506±0.0023	0.491
FATHMM-MKL	0.540	0.540±0.0013	0.555
SilVA	0.527	0.527±0.0009	
TraP	0.495	0.496±0.0038	
DDIG-SN	0.535	0.535±0.0012	

*Test set was sampled 20 times (each with 100,000 observed and 100,000 generated variants) to produce averages and standard deviations (SD) of AUCs for sSNVs.

### Predictor Performance Is Only Slightly Better for nsSNVs Than for sSNVs

**Figure 4:**
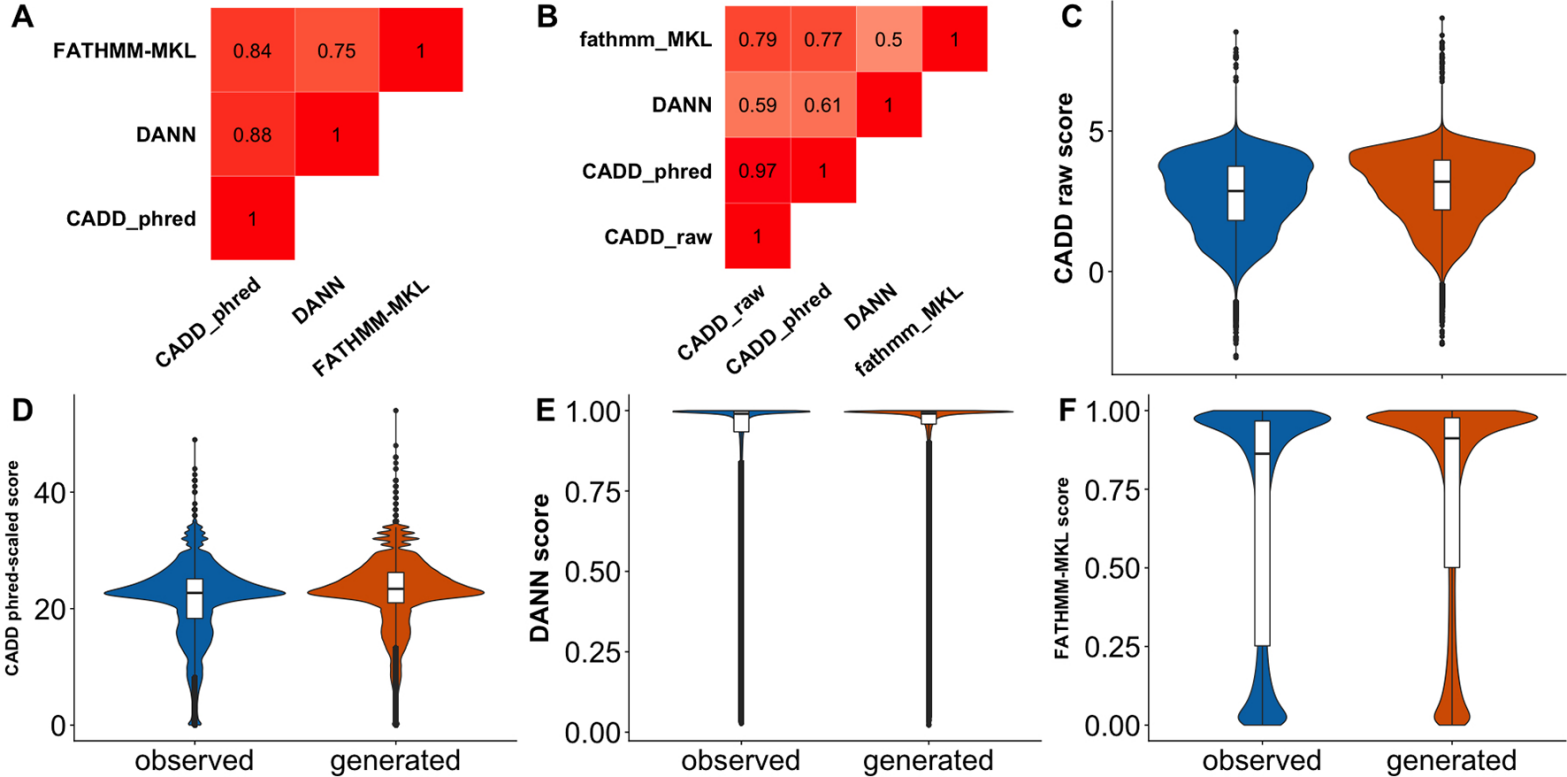
Predictor scores correlate, but do not clearly differentiate *observed* vs. *generated* nsSNVs.****Panel **(A)** shows the amount of agreement (i.e., FCBP) for any pair of predictors. High FCBP values indicate that two predictors agree in assigning binary (neutral/deleterious) predictions to variants. Panel **(B)** shows the Pearson correlations among the prediction scores. **(C**–**F)** Violin/box plots of prediction score distributions across predictors: CADD raw, CADD phred-scaled, DANN, and FATHMM-MKL, respectively.

As mentioned previously, the unexpected inability of predictors (**Figure 3**) to differentiate *observed* and *generated* variants may indicate either the inappropriateness of the data set for the evaluation task or limited predictor abilities. The latter may be related to the specific variant type; i.e., general-purpose predictors, such as CADD and FATHMM-MKL, are good at analyzing non-synonymous variants (Kircher et al., 2014; Shihab et al., 2015), but they may be less sensitive to effects of synonymous variants. To evaluate this possibility, we randomly selected 500,000 each *observed* and *generated* non-synonymous variants from dbNSFP (Liu et al., 2011; Liu et al., 2016) and extracted their associated predictor scores (see **Supplementary Material**). Briefly, an nsSNV was considered *observed* if it was reported by either 1000G, ExAC, or gnomAD; otherwise it was deemed a *generated* nsSNV. While some of the predictors were slightly better at differentiating *generated* from *observed* nsSNVs (**Figure 4**, **Table 3**) than sSNVs, their performance was still not up to the expectations. We also calculated FCBP (**Figure 4A**; cutoffs as above) and score correlation (**Figure 4B**) to find that CADD, DANN, and FATHMM-MKL have a considerably higher agreement on nsSNVs than on sSNVs (**Figure 3A**).

### Inferring a Predictor Scoring Threshold From Prediction of Common Variant Effects

**Figure 5:**
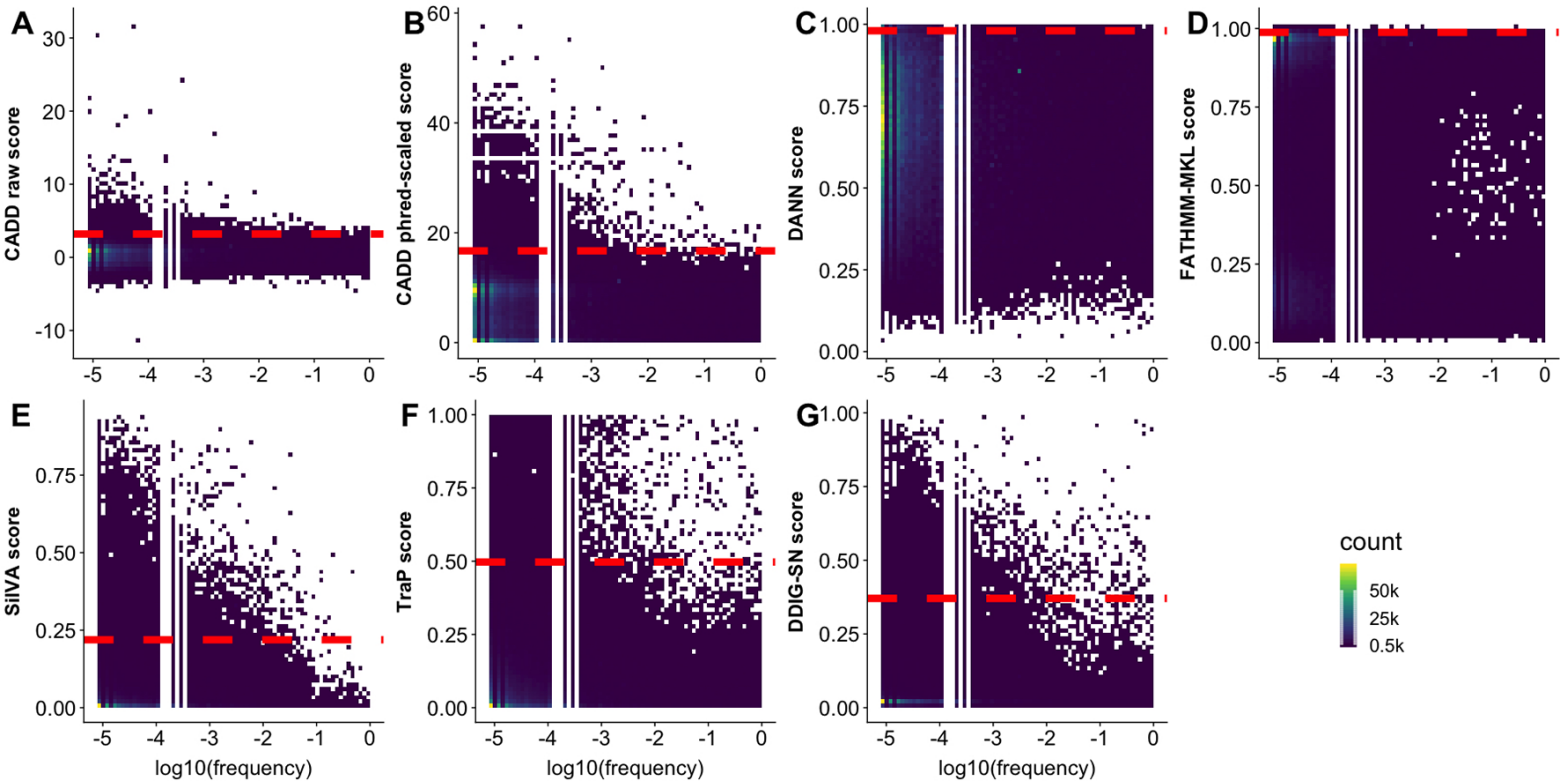
Some predictors assign higher scores to rare variants. In all panels, the scatterplots display the density of *observed* variant prediction scores *vs.* log_10_(allele frequency). A scoring threshold (red dashed line) for each predictor identifies scores above the threshold as reliable. The threshold is placed at the score that is higher than 99% of common (allele frequency > 0.01) variant scores. **(A**-**G)** represents the scatterplot for CADD raw, CADD phred-scaled, DANN, FATHMM-MKL, SilVA, TraP, and DDIG-SN, respectively.

The predictor inability to differentiate *observed* and *generated* variants may also be due to the difficulty of defining effect threshold; i.e., variants of low effect are harder to precisely annotate, both computationally and experimentally, and can be equally well classified as effect or neutral. In an effort to increase resolution between the two, predictors often link high allele frequency to absence of effect. In fact, CADD, DANN, FATHMM-MKL, SilVA, and regSNP-splicing effectively label high allele frequency variants as neutral. Taken further, TraP scores were reported (Gelfman et al., 2017) to have negative correlation (−0.51) with bin-average ExAC allele frequencies (Lek et al., 2016). Note that, as mentioned above, this reasoning side-steps evolutionary flow where common (not yet fixed or removed) variants may be advantageous or damaging. To further elaborate on allele frequency relationship with effect predictions, we obtained frequency data from multiple sources (1000G, ExAC, and gnomAD, see **Supplementary Material**) for our *observed* variants. Notably, we saw no correlation, positive or negative, between allele frequency and any predictor score (**Figure 5**). This observation highlights predictor binary classification abilities rather than a continuous spectrum of effect.

For some of the predictors (CADD, SilVA, TraP, DDIG-SN) high scoring variants were overwhelmingly of low frequency. At the same time, many of the low frequency variants were low scoring. Assuming that the predictor scores can be used as reliable indicators of common variant neutrality (low scoring), this result reinforces the idea that low frequency variants are as likely to be pathogenic/effect as neutral/benign. Furthermore, common variant score distributions could help approximate the predictor thresholds of effect. Thus, while variants scoring above a certain threshold can be considered to have an effect, below this threshold binary predictor resolution is questionable.

Predictor thresholds were chosen as the score below which most (99%) of the common variants (allele frequency >0.01) reside (**Figure 5**). Thus, scores above this threshold indicate effect, while scores below the threshold could be effect or neutral. We further applied the selected thresholds to both *observed* and *generated* sSNVs (**Table 4**). We define *resolution* (Eqn 2, where “N” stands for number) as a predictor’s ability to capture the enrichment of deleterious variants above threshold.

(2) $$resolution=\frac{N_{sSNVs\;above\;the\;threshold}}{N_{observed\;sSNVs}}\times \frac{N_{\;generated\;sSNVs}}{N_{generated\;sSNVs\;above\;the\;threshold}}$$

**Table 4:** Percentage of sSNVs scoring above threshold and the corresponding predictor resolutions.

	% Above-the-threshold sSNVs in *observed*	% above-the-threshold sSNVs in *generated*	Resolution
CADD raw score	0.871	1.981	2.274
CADD phred-scaled score	0.868	1.979	2.280
DANN	1.594	2.156	1.352
FATHMM-MKL	1.639	2.522	1.538
SilVA	4.902	6.015	1.227
TraP	2.376	2.912	1.226
DDIG-SN	1.764	2.414	1.368

The *resolutions* were greater than one for all the predictors, with CADD attaining the highest resolution (> 2). Note that only a small fraction of variants in both sets scored above the threshold, but since the total number of *generated* variants is nearly 18 times higher than the number of *observed* variants, the estimated number of potential identifiably-deleterious sSNVs is only an order of magnitude less than ALL observed sSNVs (∼475K vs. ∼1.3M). These results suggest that the *generated* set indeed contains many more deleterious variants than the *observed* set and that a new predictor train to recognize these differences may identify deleterious variants more reliably than existing methods.

## Conclusion

Training data is perhaps the most critical component for a machine learning-based variant-effect-predictor. Most sSNV effect predictors we reviewed, retrieved training data from disease mutation databases, such as HGMD and ClinVar. Disease-causing variants can be thought of as severely functionally deleterious, although non-disease variants could also be deleterious or beneficial. Moreover, identifying an sSNV as disease causing, as opposed to associated with disease, is extremely difficult, if not impossible. In fact, studies have revealed flaws of existing disease mutation databases, which may further undermine the reliability of the contained data. Progress in saturation genome mutagenesis may improve data availability in the near future. Currently, however, there is no publicly available, sufficiently large collection of variants with experimentally validated effect annotations that can be used for building a generalizable sSNV effect-predictor.

The lack of gold standard data also prevents proper evaluation of the predictors. Here, we proposed a *Test set* of *observed *and *generated* sSNVs. We assumed that the *generated* set is enriched for deleterious sSNVs due to purifying selection and expected the predictors to differentiate these from the *observed* variants. However, the predictor performance on this data was below our expectations. Note that predictor scores for the variants in our set were poorly correlated and the amount of binary prediction agreement was limited. This observation suggests that predictions may be biased by shared input features, but do not sufficiently well indicate variant effect. We proposed a scoring threshold to separate reliable predictions from the highly uncertain ones for each of the predictor. With the thresholds identified, we further observed that all predictors had significantly more reliably identified sSNVs in the *generated* set than in *observed* set, in line with our earlier expectations of the quality and contents of the *Test* set. However, the inability of the predictors to clearly identify effect variants below the severity threshold, suggests that more work is necessary to understand sSNV effects.

We note that our *Test set* is not a gold-standard testing set and is only appropriate for predictor testing only if our underlying biological/data distribution assumptions hold. Thus, we cannot make concrete recommendations of best-practice prediction tools. However, our results clearly indicate that the predictions are highly correlated across sSNV-specific methods, i.e., there is little difference between using SilVA, DDIG-SN, or TraP. On the other hand, outputs of general purpose-predictors (CADD, DANN, and FATHMM-MKL) do not correlate as well. Of these, CADD phred-scaled scores are least likely to classify common variants as having a large effect; i.e., CADD high scores may be deemed reliably non-neutral. Note, however, that this does not mean that CADD low scores indicate variant neutrality – a necessary distinction that evades much of the variant effect literature.

Looking forward to a future sSNV effect-predictor, we note that comparing *observed* vs. *generated*, rather than effect vs. no-effect, variants drastically increases the amount of data useful for training. We also note that this variant collection will need further filtering to address the problem of false positives, i.e., the yet-to-be-*observed generated* variants. Moreover, the transition from *observed* to no-effect and from *generated* to effect annotations will not be trivial. As mentioned earlier, while severe effect variants are likely predominantly confined to the *generated* set, the mild effect variation is probably distributed throughout both *observed* and *generated* collections. Despite these difficulties, the observation that existing predictors identify more higher-scoring effect variants in the *generated* data, suggests that the effect signal can indeed be learnable by models trained to differentiate *observed* vs *generated* variants. Thus, a model using the previously mentioned set of features, possibly in combination with an ensemble of (orthogonal, as evaluated above) existing classifiers, may provide a more reliable description of variant effects.

## Data Availability Statement

Public available datasets were analyzed in this study. Human transcripts and genomic coordinate information (GRCh37) can be found at https://grch37.ensembl.org/biomart/martview/e1515959acf51b72adec3001b7e02e59. DANN scores can be found at https://cbcl.ics.uci.edu/public_data/DANN/. TraP scores can be found at http://innovation.columbia.edu/technologies/cu17233_pathogenicity-database-for-identification-of-disease-causing-non-coding-genetic-variations. FATHMM-MKL scores can be found at https://github.com/HAShihab/fathmm-MKL. ANNOVAR annotation tool can be found at http://annovar.openbioinformatics.org/en/latest/. dbNSFP annotation tool can be found at https://sites.google.com/site/jpopgen/dbNSFP/. DDISN-SN server is at http://sparks-lab.org/ddig/. SilVA predictor is at http://compbio.cs.toronto.edu/silva/. MutationTaster2 server is at http://www.mutationtaster.org/StartQueryEngine.html. IDSV can be found at http://bioinfo.ahu.edu.cn:8080/IDSV. Our observed/generated data with predicted scores can be downloaded at https://doi.org/10.5281/zenodo.3471642.

## Author Contributions

ZZ and YB contributed to the idea conception, analysis design, literature review, and manuscript writing. ZZ conducted data collection, analysis, and visualization.

## Funding

ZZ and YB Were Supported by the NIH U01 GM115486 Grant.

## Conflict of Interest

The authors declare that the research was conducted in the absence of any commercial or financial relationships that could be construed as a potential conflict of interest.
