# Supplementary material for: Predicting Functional Effects of Synonymous Variants: A Systematic Review and Perspectives
Source: Front Genet. 2019 Oct 7;10:914. doi: 10.3389/fgene.2019.00914 (PMC6791167; doi:10.3389/fgene.2019.00914)
Supplement: Supplementary file 1 [file Table_1.docx]

Supplementary Material

# Assigning sSNV class (*observed* and *generated*) and allele frequency

We downloaded all human protein coding transcripts (and their genomic coordinates) from Ensembl BioMart (Kinsella, et al., 2011) GRCh37 p.13 assembly (December 2017) (Church, et al., 2011). The detailed procedures are as follows: 1) On Ensembl BioMart (<https://grch37.ensembl.org/biomart/martview/e1515959acf51b72adec3001b7e02e59>), select “Ensembl Genes” database and “Human Genes (GRCh37 p.13)” dataset; 2) select “protein_coding” as “Transcript type” under “Filters” section; 3) For “Attributes”, select “Transcript stable ID”, “CDS start”, “CDS end”, “Genomic coding start”, “Genomic coding end”, “Strand”, “Chromosome/scaffold name”, and “Coding sequence”.

After removing the problematic ones (with unknown nucleotides, no start/stop codon, etc.), we retained 93,444 transcripts. Based on the sequences of these transcripts, we *generated* all possible synonymous single nucleotide variants (sSNVs) and mapped them to genomic coordinate locations. Among these, we further selected existing sSNVs (Fig. S1) and annotated these with allele frequencies using four sequencing projects (1000 Genomes Project (Birney and Soranzo, 2015), ExAC (Lek, et al., 2016), gnomAD exome and genome (Karczewski, et al., 2019)) using ANNOVAR (Wang, et al., 2010). Note that the cohort sizes of the sequencing projects are different (1000 Genomes Project = 2,504 individuals, ExAC = 60,706, gnomAD exome = 125,748, and gnomaAD genome = 15,708). We thus assigned the final sSNV allele frequency on the basis of the largest applicable cohort. Singleton (i.e. only one variant found in the entire cohort) sSNVs were further validated or removed using sequentially smaller cohorts. The sSNV label (*observed* or *generated*) was assigned using allele frequency (Fig. S1).


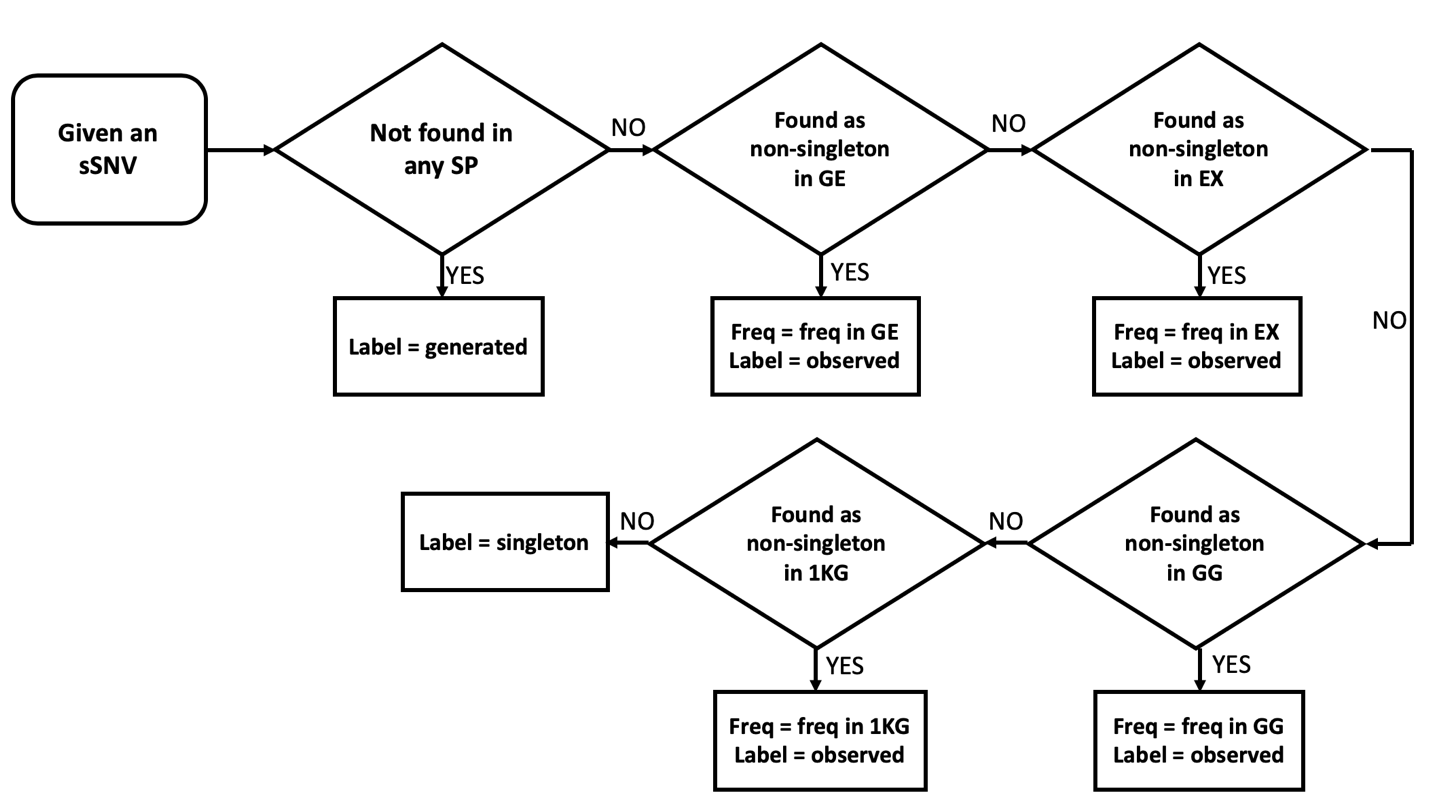
 **Supplementary Figure 1:** **Assigning sSNV labels**. Abbreviations: SP, sequencing project; *freq*, allele frequency; *GE*, gnomAD exome; *EX*, ExAC; *GG*, gnomAD genome; *1KG*, 1000 Genomes Project.

We thus retained 1,362,607 genomic coordinate-based (5,496,017 transcript-based) *observed* sSNVs and 24,008,961 coordinate-based (64,693,078 transcript-based) *generated* sSNVs. We randomly selected 50,000 *observed* and 50,000 *generated* sSNVs for preliminary analysis (Fig. S2). We included all *observed* sSNVs and equal amount (1,362,607) of randomly selected *generated* sSNVs for a comprehensive analysis described in text.


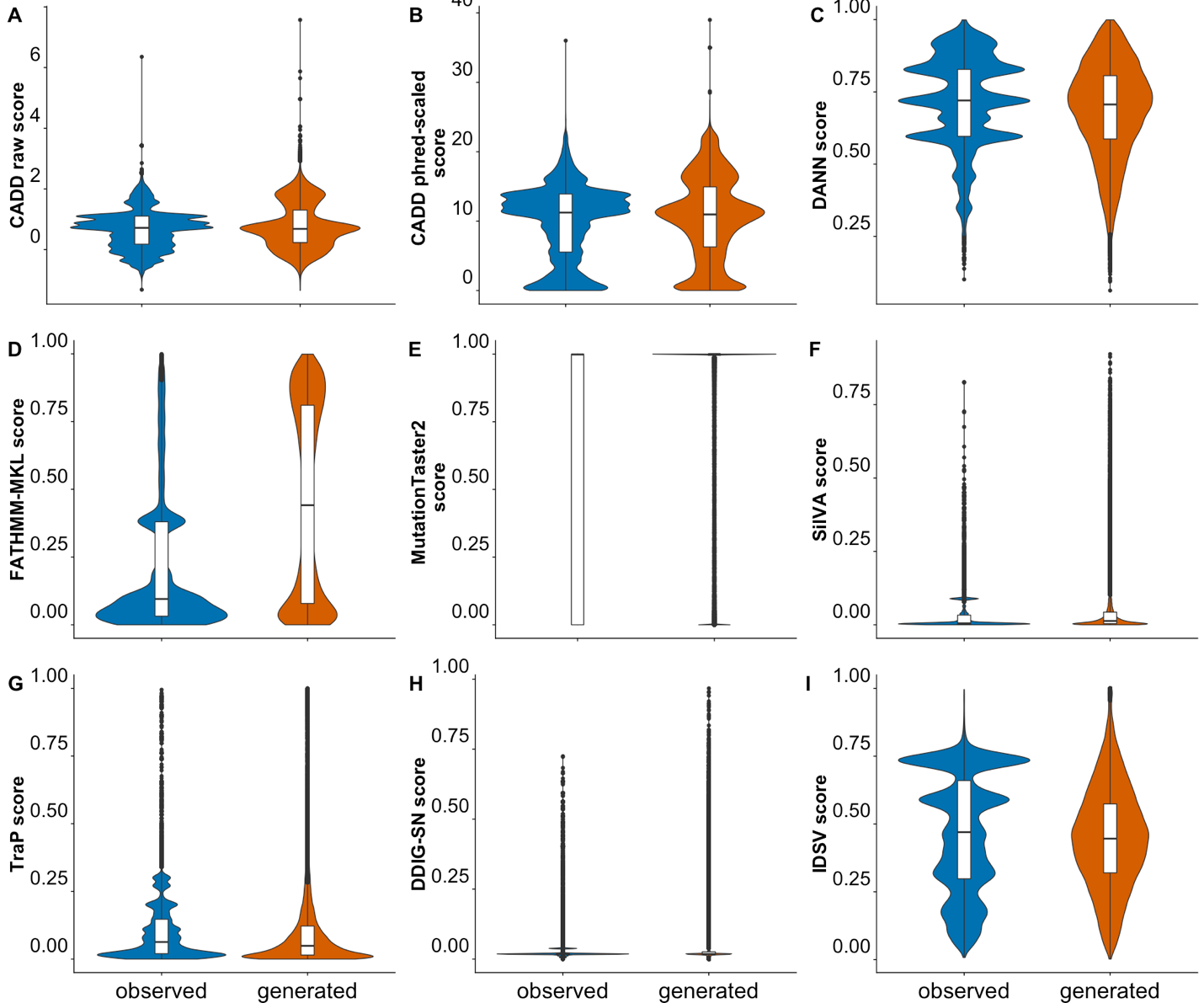


**Supplementary Figure 2. Preliminary results of predicted scores in violin plots with 50,000 *observed* and 50,000 *generated* sSNVs.** Panel A-I represent CADD raw score, CADD phred-scaled score, DANN score, FATHMM-MKL score, MutationTaster2 score, SilVA score, TraP score, DDIG-SN score, and IDSV score, respectively.

# Variant effect prediction

We included CADD (Kircher, et al., 2014), DANN (Quang, et al., 2014), MutationTaster2 (Schwarz, et al., 2014), FATHMM-MKL (Shihab, et al., 2015), SilVA (Buske, et al., 2013), DDIG-SN (Livingstone, et al., 2017), TraP (Gelfman, et al., 2017) , and IDSV (Shi, et al., 2019) predictions for our analysis. Unfortunately, at the time of writing, regSNP-splicing (Zhang, et al., 2017) prediction server was not functional and thus could not be considered.

CADD. We used ANNOVAR (Wang, et al., 2010) to retrieve CADD raw scores and phred-scaled scores for our variants.

DANN. We annotated our variants using the pre-computed DANN scores from the online repository (<https://cbcl.ics.uci.edu/public_data/DANN/>).

FATHMM-MKL. We annotated our variants using the pre-computed database from the online repository (<https://github.com/HAShihab/fathmm-MKL>).

MutationTaster2. We submitted our variants for annotation to the online server (<http://www.mutationtaster.org/StartQueryEngine.html>) with the following filters: homozygous in 1 or more TGP (1000 Genomes Project) samples, heterozygous in 1 or more TGP samples, minimum coverage = 0, analyse complete VCF only with 0 bases intron flanking.

SilVA. We downloaded SilVA (<http://compbio.cs.toronto.edu/silva/>) and ran our variant analysis locally.

DDIG-SN. We submitted our variants for annotation to the online server (<http://sparks-lab.org/ddig/>).

TraP. We annotated our variants using the pre-computed database from the online repository (<http://innovation.columbia.edu/technologies/cu17233_pathogenicity-database-for-identification-of-disease-causing-non-coding-genetic-variations>).

IDSV. The scores for our variants were kindly provided by the author (Dr. Junfeng Xia).

# Collection of observed and generated nsSNVs

We downloaded the dbNFSP database (Liu, et al., 2011; Liu, et al., 2013) (version 4.0a, <https://sites.google.com/site/jpopgen/dbNSFP>) and randomly selected 500,000 observed non-synonymous single nucleotide variants (nsSNVs) and 500,000 generated nsSNVs (hg19 genomic coordinates), fully annotated with CADD raw scores, CADD phred-scaled scores, DANN scores, and FATHMM-MKL scores. The observed/generated labels were determined by the procedure described above for sSNVs (Fig. S1).

# Collection of CADD observed and simulated sSNVs

We compared the CADD raw scores and phred-scaled scores for our -*observed* and -*generated* vs. CADD -observed and -generated (simulated) sSNVs (Fig.S3). The 51,517 CADD observed and 66,369 *generated* (simulated) training variants were kindly provided by the author (Dr. Kircher Martin). We re-annotated these sSNVs with CADD raw scores and phred-scaled scores using ANNOVAR. For simplicity of evaluation, we randomly selected equal numbers (51,517) of variants from larger data sets (CADD-generated, our *observed* and *generated* sSNVs) to match the number of CADD observed variants (Fig. S3). The ROC AUCs of *observed* vs. *generated* and CADD-observed vs. CADD-simulated were 0.518 and 0.614, respectively. Note that, as expected, CADD is able to better differentiate its own training samples, although performance on both data sets was not overly promising.


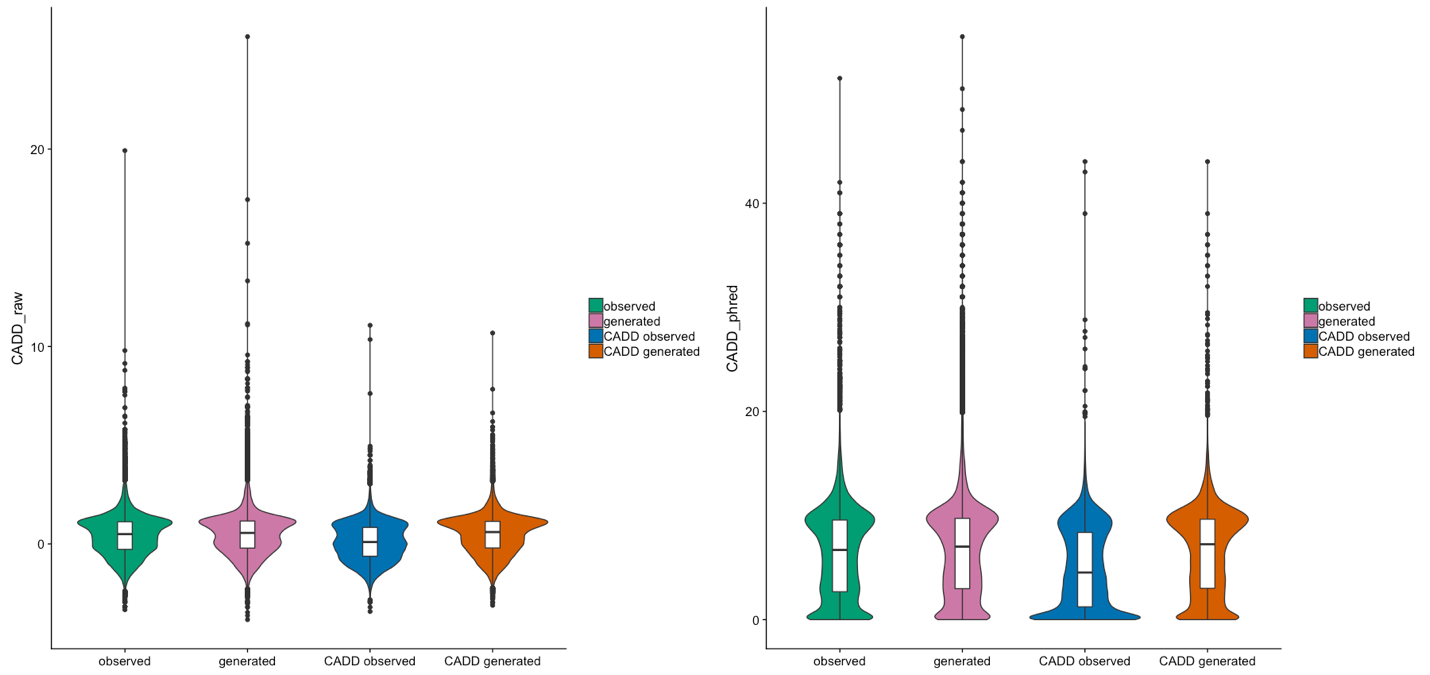


**Supplementary Figure 3**. **CADD scores on *Test set* and on CADD’s training data**. The left and right panel are CADD raw scores and phred-scaled scores, respectively.

# Data analysis and visualization

Data analysis was done in Python 3.6.4 with NumPy 1.16.2 (Van Der Walt, et al., 2011) and Pandas 0.24.1 (McKinney, 2010). Visualization was done in R (Team, 2019) with R package ggplot2 (Wickham, 2016).

# References

Birney, E. and Soranzo, N. Human genomics: The end of the start for population sequencing. 2015;526(7571):52-53.

Buske, O.J.*, et al.* Identification of deleterious synonymous variants in human genomes. *Bioinformatics* 2013;29(15):1843-1850.

Church, D.M.*, et al.* Modernizing Reference Genome Assemblies. 2011;9(7):e1001091.

Gelfman, S.*, et al.* Annotating pathogenic non-coding variants in genic regions. *Nature communications* 2017;8(1):236.

Karczewski, K.J.*, et al.* Variation across 141,456 human exomes and genomes reveals the spectrum of loss-of-function intolerance across human protein-coding genes. *bioRxiv* 2019:531210.

Kinsella, R.J.*, et al.* Ensembl BioMarts: a hub for data retrieval across taxonomic space. 2011;2011(0):bar030-bar030.

Kircher, M.*, et al.* A general framework for estimating the relative pathogenicity of human genetic variants. *Nature genetics* 2014;46(3):310.

Lek, M.*, et al.* Analysis of protein-coding genetic variation in 60,706 humans. *Nature* 2016;536(7616):285-291.

Liu, X., Jian, X. and Boerwinkle, E. dbNSFP: A lightweight database of human nonsynonymous SNPs and their functional predictions. *Human Mutation* 2011;32(8):894-899.

Liu, X., Jian, X. and Boerwinkle, E. dbNSFP v2.0: A Database of Human Non-synonymous SNVs and Their Functional Predictions and Annotations. 2013;34(9):E2393-E2402.

Livingstone, M.*, et al.* Investigating DNA‐, RNA‐, and protein‐based features as a means to discriminate pathogenic synonymous variants. *Human mutation* 2017;38(10):1336-1347.

McKinney, W. Data structures for statistical computing in python. In, *Proceedings of the 9th Python in Science Conference*. Austin, TX; 2010. p. 51-56.

Quang, D., Chen, Y. and Xie, X. DANN: a deep learning approach for annotating the pathogenicity of genetic variants. *Bioinformatics* 2014;31(5):761-763.

Schwarz, J.M.*, et al.* MutationTaster2: mutation prediction for the deep-sequencing age. *Nature methods* 2014;11(4):361.

Shi, F.*, et al.* Computational identification of deleterious synonymous variants in human genomes using a feature-based approach. *BMC medical genomics* 2019;12(1):12.

Shihab, H.A.*, et al.* An integrative approach to predicting the functional effects of non-coding and coding sequence variation. *Bioinformatics* 2015;31(10):1536-1543.

Team, R.C. R: A language and environment for statistical computing. Vienna, Austria: R Foundation for Statistical Computing. 2018. *Google Scholar* 2019.

Van Der Walt, S., Colbert, S.C. and Varoquaux, G. The NumPy array: a structure for efficient numerical computation. *Computing in Science & Engineering* 2011;13(2):22.

Wang, K., Li, M. and Hakonarson, H. ANNOVAR: functional annotation of genetic variants from high-throughput sequencing data. *Nucleic Acids Research* 2010;38(16):e164-e164.

Wickham, H. ggplot2: elegant graphics for data analysis. Springer; 2016.

Zhang, X.*, et al.* regSNPs-splicing: a tool for prioritizing synonymous single-nucleotide substitution. *Human genetics* 2017;136(9):1279-1289.
